# Influence of Deep Neuromuscular Blockade on Perioperative Stress Response in Patients Undergoing Robot-Assisted Gastrectomy: A Prospective Double-Blinded Randomized-Controlled Trial

**DOI:** 10.3390/jpm11121308

**Published:** 2021-12-06

**Authors:** Myoung Hwa Kim, Na Young Kim, Young Chul Yoo, Hee Jung Kong, Hye Sun Lee, Arim Jo, Sun Joon Bai

**Affiliations:** 1 Department of Anesthesiology and Pain Medicine, Anesthesia and Pain Research Institute, Yonsei University College of Medicine, Gangnam Severance Hospital, 211 Eonju-ro, Gangnam-gu, Seoul 06273, Korea; kmh2050@yuhs.ac (M.H.K.); ARIMJO@yuhs.ac (A.J.); 2 Department of Anesthesiology and Pain Medicine, Anesthesia and Pain Research Institute, Yonsei University College of Medicine, 50-1 Yonsei-ro, Seodaemun-gu, Seoul 03722, Korea; knnyyy@yuhs.ac (N.Y.K.); seaoyster@yuhs.ac (Y.C.Y.); sarajo8768@yuhs.ac (H.J.K.); 3 Department of Research Affairs, Biostatistics Collaboration Unit, Yonsei University College of Medicine, 50-1 Yonsei-ro, Seodaemun-gu, Seoul 03722, Korea; HSLEE1@yuhs.ac

**Keywords:** neuromuscular blockade, robotic surgery, stress response, postoperative recovery

## Abstract

Attenuating the intraoperative stress response is crucial; however, the effect of neuromuscular blockade (NMB) on surgical stress is not completely understood. We aimed to investigate the effects of NMB on the perioperative stress response during robot-assisted gastrectomy. Patients were assigned to the deep or moderate NMB group. Serum cortisol, prolactin, and interleukin-6 (IL-6) levels and natural killer (NK) cell percentage were measured before anesthesia induction, 90 min after pneumoperitoneum, operation end (OP_End_), and postoperative day 1. Additionally, C-reactive protein (CRP) and albumin levels were estimated. Additionally, intraoperative heart rate variability was evaluated. The deep NMB group showed significantly lower levels of low-frequency/high-frequency (HF) ratio at OP_End_ compared to the moderate NMB group (1.4 ± 0.2 vs. 2.2 ± 0.3, respectively; Bonferroni corrected *p* = 0.039). Furthermore, HF power in the deep NMB group was significantly higher at OP_End_ than that in the moderate NMB group (45.2 ± 3.6 vs. 33.8 ± 4.0, respectively; Bonferroni corrected *p* = 0.044). However, no significant differences in cortisol, prolactin, IL-6, CRP, and albumin levels and NK cell percentage were found between the two groups. The degree of NMB may have similar effects on stress-related biological markers in patients undergoing robot-assisted gastrectomy.

## 1. Introduction

Stomach cancer is a major cause of cancer-associated deaths worldwide, and surgical resection is considered the most effective treatment strategy [1,2]. Recently, robot-assisted gastrectomy has been increasingly regarded as a minimally invasive alternative. However, this technique requires carbon dioxide (CO_2_) pneumoperitoneum over 12 mmHg and recommends patients to be in the head-up position for the duration of the surgery for better surgical view, both of which are predisposing conditions for autonomic nervous system imbalance. This situation causes stimulation of sympathetic activity, aggravating the perioperative stress response [3,4,5].

Despite removal of the gross mass, there is a risk of cancer micro-metastasis due to surgical manipulation or contact between the cancer cells and bloodstream [6]. In immunocompetent patients, this micro-metastasis is usually cleared within 24 h. However, perioperative stress can induce the release of stress hormones, such as catecholamines and glucocorticoids, which attenuate the activity of the immune system and temporarily compromise the immune function for up to 7 days after surgery [7]. Therefore, attenuating the intraoperative stress response is especially important in cancer surgeries, as the patients have a compromised immune function [8,9].

Deep neuromuscular blockade (NMB) enhances surgical conditions by facilitating relaxation of the abdominal wall and preventing abrupt muscle contraction. The use of NMB enhances surgeon satisfaction during laparoscopic surgery involving the pneumoperitoneum, even during low-pressure laparoscopic surgeries [10,11,12]. Deep NMB facilitates robotic surgery in a state of low intra-abdominal pressure, which may reflect the attenuation of sympathetic hyperactivity [13]. There are several reports on the methods of attenuating the stimulation of the sympathetic activity that could potentially aggravate the stress response [13,14]. However, reports regarding the effect of deep NMB itself on the stress response are lacking.

Considering the advantages of deep NMB reported previously, we expect deep NMB to potentially minimize tissue injuries, maintain autonomic nervous system balance, and improve oncologic immunity in those undergoing robot-assisted gastrectomy. Therefore, we aimed to investigate the effects of deep NMB, compared with moderate NMB, on the perioperative stress response in patients undergoing robot-assited gastrectomy. Furthermore, we evaluated the intraoperative balance of the autonomic nervous system.

## 2. Materials and Methods

### 2.1. Study Population

This prospective, randomized, double-blinded clinical trial was approved by the Institutional Review Board (Institutional Review Board number: 4-2019-0205) of Severance Hospital, Yonsei University Health System (Seoul, Republic of Korea) and registered at clinicaltrials.gov (ClinicalTrial.gov, NCT03937440, 5 March 2019). This study was performed in accordance with relevant guidelines and regulations. A total of 46 patients with an American Society of Anesthesiologists physical status classification of I or II, aged between 20 and 65 years, and scheduled for robot-assisted gastrectomy between May and December 2019 were included. Patients with neuromuscular disease; those receiving β-blockers for hypertension or insulin therapy for diabetes mellitus; and those with heart failure, end-stage renal disease, severe hepatic dysfunction (alanine aminotransferase >3 times the normal upper limit), or morbid obesity were excluded. Vulnerable subjects who were unable to provide consent were also excluded. Written informed consent was obtained from each patient.

### 2.2. Study Design and Intervention

A computer-generated randomization table was used to randomly allocate patients to the deep (*n* = 23) or moderate NMB group (*n* = 23) in a 1:1 ratio, and copies of the random sequence were kept in sealed, opaque envelopes. Randomization was not blocked or stratified. In the deep NMB group, 1 mg/kg of rocuronium was administered for facilitating tracheal intubation. Subsequently, rocuronium infusion was initiated at a rate of 0.3 mg/kg/h, which was adjusted to maintain a post-tetanic count of 1–2 [15,16]. In the moderate NMB group, after administration of 0.6 mg/kg of rocuronium for intubation, rocuronium infusion was started at a rate of 0.2 mg/kg/h, which was regulated to maintain a target of 1–2 train-of-four (TOF) [15,16]. The investigators who assessed the postoperative outcomes, patients, and surgeons were all blinded to the group assignment.

### 2.3. Anesthetic Management

All patients were monitored with electrocardiography, pulse oximetry, and non-invasive blood pressure before the induction of anesthesia. A bispectral index (BIS) monitor (BIS^TM^ QuatroSensor, Covidien, Mansfield, MA, USA) was applied to the forehead of patients for monitoring the depth of anesthesia. Anesthesia was induced with intravenous propofol 1–2 mg/kg and remifentanil 0.1 μg/kg/min. Two electrodes were attached to the forearm along the path of the ulnar nerve, and a transducer was fixed on the thumb to monitor the twitching of the adductor pollicis muscle. The transducer was connected to an acceleromyograph (TOF-Watch^®^ SX, Organon Ltd., Drynam Road, Swords, Co. Dublin, Ireland). Rocuronium (Esmeron™, N.V. Organon, Oss, The Netherlands) was injected for neuromuscular relaxation according to the group allocation: 1 mg/kg in the deep NMB group and 0.6 mg/kg in the moderate NMB group. After endotracheal intubation, the radial artery was catheterized for continuous blood pressure monitoring and blood sampling. Anesthesia was maintained by desflurane inhalation at 4–6 vol% and remifentanil infusion at 0.05–0.2 μg/kg/min; the mean arterial pressure was maintained within 20% of the baseline value, and a BIS of 40–60 was maintained.

Robot-assisted gastrectomy was conducted via the da Vinci Surgical System (Intuitive Surgical, Sunnyvale, CA, USA) [10,11]. The patients were positioned in the reverse Trendelenburg position at 15–20°. The camera port was inserted with a 12 mm trocar in the infra-umbilical area. The initial intra-abdominal pressure was 12 mmHg in both groups, and it was maintained throughout the procedure. Three 8 mm trocars were inserted in the subcostal area, left subcostal area, and right paraumbilical area. One assistant port was placed with a 12 mm trocar in the left paraumbilical area. Intraoperative administration of the neuromuscular blocking agent was performed according to the assigned group. Continuous infusion of rocuronium was terminated after the completion of CO_2_-induced pneumoperitoneum. After desufflation of the CO_2_ pneumoperitoneum, the patients were placed in the supine position. Surgeon satisfaction regarding the surgical conditions was postoperatively assessed in both groups using a numerical rating scale (1 = least satisfied to 5 = most satisfied). The NMB of all patients was reversed using sugammadex at 2 mg/kg and 4 mg/kg for TOF ratios 2 and 0, respectively.

### 2.4. Outcome Assessments

The primary outcome was the perioperative stress response. Therefore, we examined the levels of stress response-related biological markers, including cortisol, prolactin, and interleukin-6 (IL-6) levels and natural killer (NK) cell percentage values in the lymphocytes of whole blood. For these hematological examinations, approximately 12 mL of the patient’s blood was collected at four different time points: before induction of anesthesia (baseline), 90 min after pneumoperitoneum in the head-up position, end of the operation, and postoperative day 1 (POD1). The blood samples were analyzed by the Department of Laboratory Medicine as follows: cortisol levels were measured using VITROS XT 7600 with cortisol (Ortho-Clinical Diagnostics, Raritan, NJ, USA) through a competitive immunoassay technique, and prolactin levels were measured using Atellica IM 1600 Analyzer with Atellica IM prolactin (Siemens, Erlangen, Germany) via a chemiluminescence 2-site sandwich immunoassay. IL-6 levels were estimated using Cobas e 801 with Elecsys IL-6 (Roche diagnostics GmbH, Rotkreuz, Switzerland) through an electrochemiluminescence immunoassay, and NK cell percentage was measured using NAVIOS EX (COULTER, Indianapolis, IN, USA) via a flow cytometer. In addition, C-reactive protein (CRP) and albumin levels were measured pre-operation (PreOP) and on PODs 0, 1, 2, 3, and 5.

Secondary outcomes included the balance of the autonomic nervous system as measured by heart rate variability (HRV), hemodynamic variables, and postoperative recovery profiles. Standard real-time automated three-lead electrocardiography was continuously performed, and the LabChart Pro v8 data acquisition system with HRV transducers (AD Instrument, Co., Dunedin, New Zealand) analyzed HRV for the evaluation of autonomic nervous balance. For each time point, consecutive 5 min data segments without ectopic beats or artifacts were selected and analyzed. In the frequency domain analyses, two main power spectrum components were collected: the high-frequency (HF) power spectrum (HF: 0.15–0.4 Hz) and low-frequency (LF) power spectrum (LF: 0.04–0.15 Hz). Parasympathetic activity is associated with the HF range, whereas sympathetic activity is associated with the LF range of heart rate modulation [17]. The LF/HF power ratio was also calculated to evaluate autonomic nervous system balance.

Postoperative recovery profiles including surgical pain, the use of rescue analgesics, the presence of nausea, the use of antiemetics, bowel function recovery, complications, and length of hospital stay were documented. The pain intensity was assessed using a numerical rating scale (NRS; 0, no pain, and 10, worst imaginable pain) [18] at 1 h, 6 h, 24 h, and 48 h after surgery, when the patient was resting and when they were active.

### 2.5. Statistical Analyses

Sample size was obtained based on previous studies [13,14], which used cortisol to assess the difference in perioperative stress response between the two groups. The mean difference between the baseline cortisol level and the highest cortisol level during surgery was 8.3 µg/dL, with a standard deviation of 6.2 µg/dL. We estimated that 19 patients would be required in each group, with 80% power at a significance level (alpha) of 0.05 using a two-sided two-sample unequal-variance *t*-test. Factoring in a 20% dropout rate, we enrolled 23 subjects in each group.

Student’s *t*-test or the Mann–Whitney test was used for continuous variables, and the Chi-squared test or Fisher’s exact test was performed to analyze frequency variables. Continuous variables with repeated measures, such as cortisol, prolactin, and IL-6 levels; NK cell percentage; values of CRP and albumin; blood pressure; pulse rate; and HRV data were analyzed using the linear mixed model with the patient indicator as a random effect and group, time, and group-by-time as fixed effects. We also conducted post hoc analyses and applied the Bonferroni correction for multiple comparisons. Statistical analyses were performed using SAS (version 9.4, SAS Inc., Cary, NC, USA). Differences were considered significant at a *p*-value of <0.05.

## 3. Results

### 3.1. Patients

**Figure 1:**
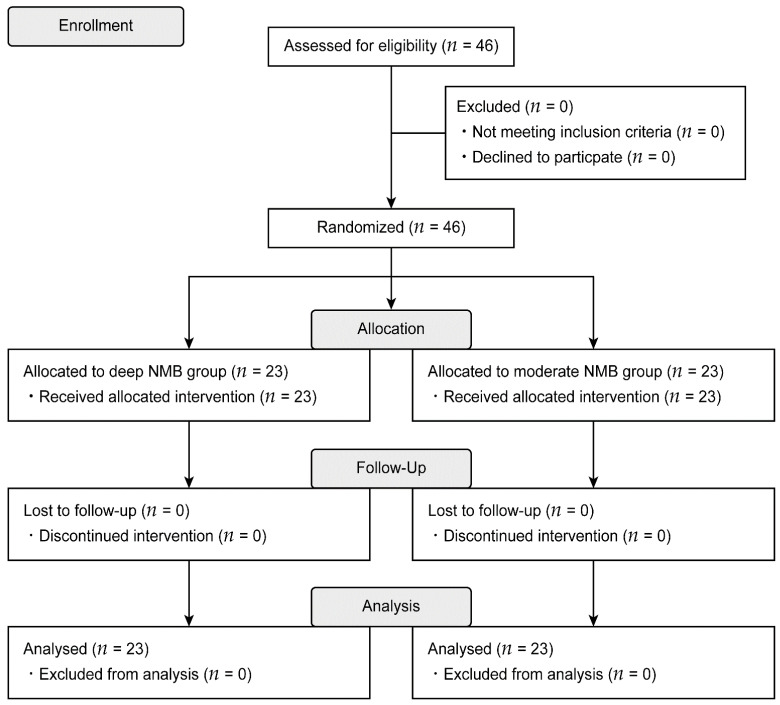
CONSORT diagram. NMB, neuromuscular blockade.

Of the 46 patients assessed for eligibility, none met the criteria for exclusion. Thus, 46 patients were enrolled, and each patient was randomly assigned to one of the two groups. All patients in both groups completed the study and were included in the final analysis (Figure 1).

### 3.2. Demographic and Intraoperative Characteristics

Demographic data, including diagnosis and surgical procedure, were similar between the deep and moderate NMB groups. However, surgeons’ request for additional NMB was significantly more frequent in the moderate NMB group than that in the deep NMB group (*p* = 0.016) (Table 1).

### 3.3. The Intraopeartive HRV

**Figure 2:**
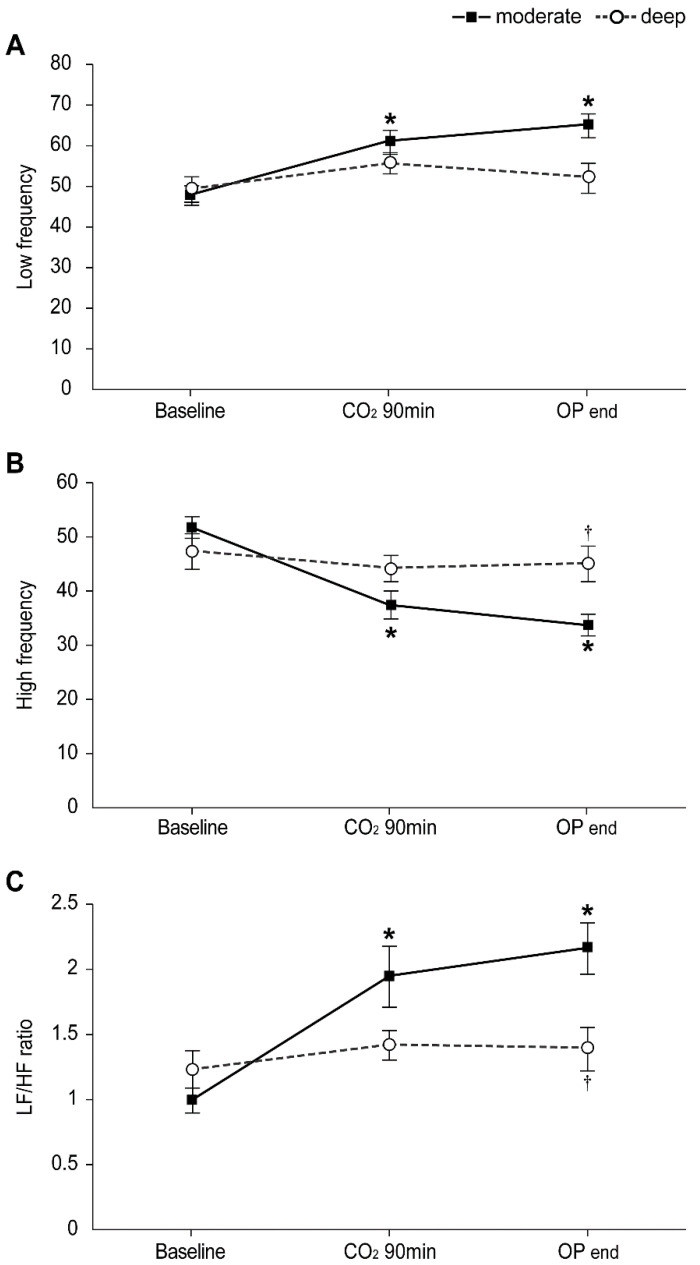
Changes in intraoperative heart rate variability (HRV) in the moderate (▪) and deep (◦) NMB groups: (**A**) Low frequency, (**B**) High frequency, (**C**) LF/HF ratio. Values are mean ± SE. HF: high frequency; LF: low frequency; Baseline: before anesthesia induction; CO_2_ 90 min: 90 min after pneumoperitoneum in the head-up position; OP end: end of operation. * Bonferroni corrected *p* < 0.05 compared with the baseline value in each group. ^†^ Bonferroni corrected *p* < 0.05 compared with the moderate NMB group.

Figure 2 demonstrates the intraoperative HRV as LF power, HF power, and LF/HF ratio, which were comparable between the groups before induction of anesthesia. Following linear mixed model analysis, significant intergroup differences in the LF/HF ratio were shown over time between the two groups (*p*_Group × time_ = 0.025). In the moderate NMB group, the LF power and LF/HF ratio were significantly increased at 90 min after pneumoperitoneum and at the end of the operation compared to the baseline value (before the induction of anesthesia), and HF power was significantly decreased, while no differences were observed in the deep NMB group. Therefore, compared to the moderate NMB group, the deep NMB group showed significantly lower levels of LF/HF ratio at the end of the operation (1.4 ± 0.2 vs. 2.2 ± 0.3, respectively; Bonferroni corrected *p* = 0.039), but no differences existed between the groups at 90 min after pneumoperitoneum (1.4 ± 0.2 in the deep NMB group vs. 1.9 ± 0.3 in the moderate NMB group). Furthermore, HF power in the deep NMB group was significantly higher at the end of the operation than that in the moderate NMB group (45.2 ± 3.6 vs. 33.8 ± 4.0, respectively; Bonferroni corrected *p* = 0.044).

### 3.4. Stress Response-Related Laboratory Markers: Cortisol, Prolactin, IL-6 Levels, and NK Cell Percentage

**Figure 3:**
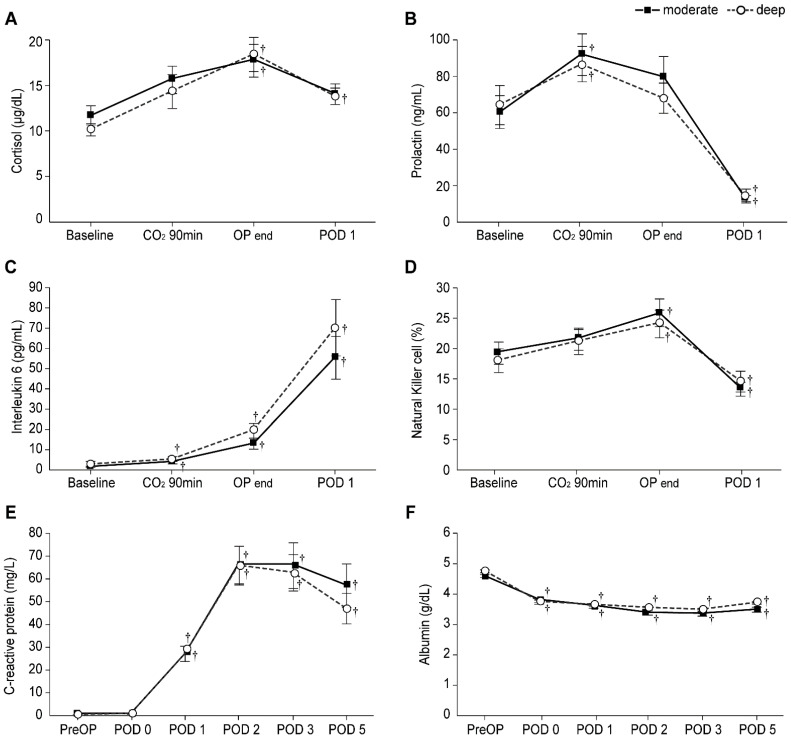
Levels of stress response-related laboratory markers in the moderate (▪) and deep (◦) NMB groups: Levels of (**A**) Cortisol, (**B**) Prolactin, (**C**) Interleukin-6, (**D**) Natural killer cells percentage, (**E**) C-reactive protein, and (**F**) Albumin. Values are mean ± SE. Baseline: before anesthesia induction; CO_2_ 90 min: 90 min after pneumoperitoneum in the head-up position; OP end: end of operation; PreOP: preoperative value; POD 0, 1, 2, 3, and 5: postoperative day 0, 1, 2, 3, and 5. ^†^ Bonferroni corrected *p* < 0.05 compared with the baseline value in each group.

Figure 3 shows the levels of stress response-related laboratory markers between the two groups. In both groups, IL-6 levels from CO_2_ 90 min to POD 1 and NK cell percentage from the end of the operation until POD 1 were found to be significantly higher than the baseline values (all Bonferroni corrected, *p* < 0.001). The prolactin levels in the two groups were significantly increased at CO_2_ 90 min (Bonferroni corrected *p* = 0.003 and 0.046 in the moderate and deep NMB groups, respectively) and significantly decreased on POD 1 (all Bonferroni corrected, *p* < 0.001) compared to the baseline values. The cortisol level in the moderate NMB group was significantly higher at the end of the operation (Bonferroni corrected *p* = 0.021), while those in the deep NMB group were significantly higher from the end of the operation until POD 1 (Bonferroni corrected *p* = 0.001 and 0.032, respectively) than the baseline values; however, no significant differences in cortisol, prolactin, and IL-6 levels and NK cell percentage were noted between the two groups. In addition, in both groups, the CRP levels from POD 1 to 5 were significantly higher (all Bonferroni corrected, *p* < 0.001), and albumin levels from POD 0 to 5 were significantly lower (all Bonferroni corrected, *p* < 0.001) than preoperative values; no significant differences in the CRP and albumin levels between the two groups were observed.

### 3.5. Intraoperative Mean Blood Pressure and Heart Rate

**Figure 4:**
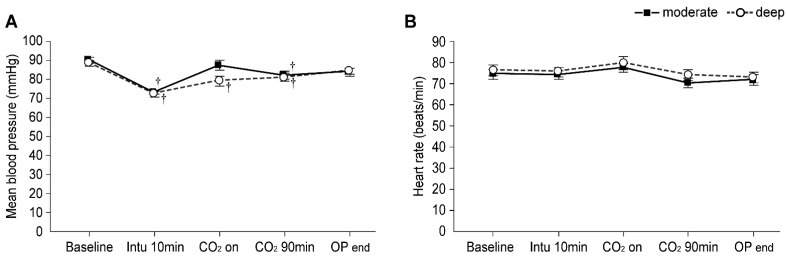
Intraoperative hemodynamics in the moderate (▪) and deep (◦) NMB groups: (**A**) Mean blood pressure and (**B**) Heart rate. Values are mean ± SE. Baseline: before anesthesia induction; CO_2_ 90 min: 90 min after pneumoperitoneum in the head-up position; OP end: end of operation. ^†^ Bonferroni corrected *p* < 0.05 compared with the baseline value in each group.

Intraoperative hemodynamics are presented in Figure 4. Patients in the deep NMB group showed significantly lower mean blood pressure from 10 min after intubation until CO_2_ 90 min (Bonferroni correct *p* < 0.001, *p* = 0.017, and *p* = 0.042 at 10 min after intubation, CO_2_ on, and CO_2_ 90 min, respectively), and those in the moderate NMB group exhibited significantly lower mean blood pressure at only 10 min after intubation and CO_2_ on (Bonferroni correct *p* < 0.001 and 0.036, respectively) compared to the baseline values; however, no significant differences in the mean blood pressure were observed between the two groups. Further, no difference in heart rate were noted between the groups.

### 3.6. Postoperative Recovery Profiles

Postoperative recovery profiles, including resting and active pain intensity, the number of patients requesting for rescue analgesics and receiving additional antiemetic agents, the incidence of postoperative complications, bowel function recovery, and length of hospital stay, were not significantly different between the two groups (Table 2). However, the surgeon’s satisfaction with surgical conditions was significantly higher in the deep NMB group (*p* = 0.014). Three patients experienced pleural effusion, and one developed intraperitoneal infection in the moderate NMB group, while one patient experienced ileus and another manifested intraperitoneal infection in the deep NMB group. However, there was no statistically significant difference in the incidence of postoperative surgical complications between the two groups. All patients with postoperative complications recovered through medication, such as antibiotics, and no incidence of reoperation or admission to the intensive care unit in either group were noted.

## 4. Discussion

This prospective, randomized, double-blinded study investigated the effects of NMB on the perioperative stress response during robot-assisted gastrectomy. Based on the results of intraoperative HRV, the deep NMB group retained significantly greater autonomic nervous system balance than did the moderate NMB group; however, no significant differences were found in the levels of stress response-related biological markers including cortisol, prolactin, IL-6 levels, and NK cell percentage. In addition, postoperative recovery profiles were comparable between the deep and moderate NMB groups.

Surgical stimuli induce pathophysiological changes, including activation of the sympathetic nervous system, that alter a patient’s immunological and metabolic functions. This response subsequently activates the release of pituitary hormones and inflammatory cytokines and increases insulin resistance [19]. Furthermore, surgical stress is well known to exert deleterious effects on a patient’s prognosis through impaired wound healing, cardiovascular and respiratory complications, and immunosuppression [20]. In one study, intraoperative catecholamine concentrations were correlated with poor postoperative outcomes, such as postoperative hypertension or the occurrence of morbid cardiac events [21]. Attenuation of the sympathetic neuroendocrine response to intense noxious stimuli during surgery improved these outcomes by exerting a beneficial influence on organ function [16]. Accordingly, there has been considerable interest in altering the intraoperative stress response for its potential beneficial effects on the postoperative outcome. Therefore, we evaluated the effects of NMB on the intraoperative stress response by assessing the stress-induced hormones, inflammatory and immune markers, and autonomic nervous balance.

Surgical injury is also associated with a stress response that varies according to the nature and degree of invasive tissue damage. Critical to this response is the release of cortisol, which is mediated by the hypothalamic–pituitary–adrenal (HPA) axis [19]. Therefore, cortisol is used in the perioperative setting as a marker of surgical stress response [22,23,24], and cortisol release during elective surgery is positively correlated with surgical severity [25]. Prolactin is also associated with the regulation of stress responses via inhibition of the HPA axis. Stress stimuli activate the HPA axis, promoting the release of corticotropin-releasing hormone in the paraventricular nucleus to trigger the secretion of adrenocorticotropic hormone from the pituitary gland. Consequently, adrenocorticotropic hormone promotes the release of glucocorticoids such as cortisol from the adrenal glands. Prolactin is also secreted from the pituitary gland in response to several stress factors [26,27].

Inflammation is strongly associated with various types of cancer [28], and IL-6 is a major cytokine released in the tumor microenvironment. IL-6 concentration rapidly increases within 30–90 min after skin incision in elective surgery; therefore, it is assumed to be a sensitive marker for early tissue injury [29]. High levels of circulating IL-6 are associated with poor outcomes and shorter survival, while lower IL-6 levels are associated with a better response to treatment [30,31]. NK cells, which act in establishing anti-tumor immunity, are profoundly suppressed by surgery and remain suppressed for a few weeks postoperatively [32,33]. Furthermore, IL-6 inhibits the cytotoxic activity of NK cells, indicating a potential antagonistic role of IL-6 on NK cell function [34,35,36]. We found that the perioperative cortisol, prolactin, and IL-6 levels and NK cell percentage were not significantly different between the deep and moderate NMB groups. Thus, the degree of NMB may not directly influence the intraoperative stress response in robot-assisted gastrectomy.

The spectral analyses of HRV are frequently used as a non-invasive method to evaluate autonomic nervous system function [37,38], and they are assessed by measuring fluctuations in the R-R interval by electrocardiography [17]. Different frequency bands of the HRV power spectrum are associated with the autonomic nervous system’s sympathetic and parasympathetic activities, and the LF/HF ratio reflects the balance between the sympathetic and parasympathetic outflow in the autonomic nervous system [37]. Our results indicated that the intraoperative sympathetic–parasympathetic balance was significantly better in the deep NMB group. Hyper-activation of the sympathetic nervous system and an overall imbalance of the autonomic nervous system, which are both considered part of the stress response, were significantly more pronounced in the moderate NMB group compared to those in the deep NMB group. This implies that deep NMB could have a positive effect on the intraoperative stress response to an extent.

Postoperative recovery, including postoperative pain, bowel function, incidence of complications, and length of the hospital stay were evaluated, and no significant intergroup differences were identified. There is insufficient evidence regarding the beneficial effects of deep NMB on the postoperative outcomes. Based on our findings, it may still be too early to definitively conclude that deep muscle relaxation has a direct positive effect on the stress response or that it affects the short-term prognosis in patients who underwent robot-assisted gastrectomy. To the best of our knowledge, this is the first to investigate the effect of deep NMB on the perioperative stress response in patients undergoing robotic surgery. However, there were some limitations to this study. Firstly, more diverse biomarkers to assess the stress response should have been used, including both NK cell activity and NK cell percentage, because for even the low NK cell percentage value, its high activity could not be excluded. Additionally, as the hematological measurements were taken at only four time points (three perioperatively and one 24 h postoperatively), the potential variation in peak levels across patients may not have been considered. However, the laboratory tests require considerable financial resources. Consequently, there exists a real limitation in terms of conducting further blood sampling in a greater number of patients for multiple laboratory analyses, mandating large corresponding resources. Nevertheless, rigorous criteria were applied for subject selection to minimize external stress-inducing factors. Secondly, the surgical duration of robot-assisted gastrectomy was relatively short, and the position adopted for patients was the reverse Trendelenburg position. These factors would have less effect than the Trendelenburg position on the perioperative stress of patients. Therefore, further studies assessing other types of robot-assisted surgeries are necessary to obtain data on the direct or indirect effects of deep muscle relaxation.

## 5. Conclusions

In conclusion, based on intraoperative HRV, autonomic nervous system balance was maintained in patients under deep NMB during robot-assisted gastrectomy The stress response-related biological markers were comparable between patients under deep NMB status and patients under moderate NMB status. This study revealed that the degree of NMB may have similar effects on stress-related biological markers in pneumoperitoneum.

## Figures and Tables

**Table 1:** Patient demographics and intraoperative variables.

Variable	Moderate NMB (*n* = 23)	Deep NMB (*n* = 23)	*p* Value
Age, year	55.0 ± 8.2	52.1 ± 8.8	0.253
Male sex	10 (43%)	12 (52%)	0.555
Body mass index, kg/m^2^	23.0 ± 3.3	23.5 ± 3.0	0.553
ASA physical status			>0.999
I	4 (17%)	5 (22%)	
II	19 (83%)	18 (78%)	
Pneumoperitoneum time, min	149 ± 51	170 ± 64	0.228
Operation time, min	177 ± 47	194 ± 67	0.312
Anesthesia time, min	207 ± 48	228 ± 68	0.232
Administered dose of remifentanil, μg	708 ± 249	717 ± 296	0.918
Administered dose of rocuronium, mg	88 ± 37	133 ± 60	0.004 *
Additional request of rocuronium	9 (39%)	2 (9%)	0.016 *
Intraoperative fluid intake and outtake			
Crystalloid, mL	1339 ± 448	1378 ± 463	0.772
Colloid, mL	102 ± 192	217 ± 295	0.124
Blood loss, mL	42 ± 53	41 ± 42	0.903
Urine output, mL	196 ± 126	202 ± 138	0.872
Type of operation			0.498
Subtotal gastrectomy	18 (78%)	21 (91%)	
Proximal subtotal gastrectomy	2 (9%)	0 (0%)	
Total gastrectomy	3 (13%)	2 (9%)	
Type of reconstruction and anastomosis			0.259
Billoth I	17 (74%)	21 (91%)	
Billoth II	2 (9%)	0 (0%)	
Double tract	2 (9%)	0 (0%)	
Roux-en-Y	2 (9%)	4 (17%)	
Extent of lymph node dissection			0.500
D1	18 (78%)	17 (74%)	
D2	5 (22%)	6 (26%)	
TNM stage			0.346
I	22 (96%)	19 (83%)	
II	1 (4%)	3 (13%)	
III	0 (0%)	1 (4%)	

Values are presented as mean ± standard deviation or number of patients (percentage). ASA: American Society of Anesthesiologists; TNM: tumor-node metastasis; NMB: neuromuscular blockade. * *p* < 0.05.

**Table 2:** Postoperative recovery profiles.

Variable	Moderate NMB (*n* = 23)	Deep NMB (*n* = 23)	*p* Value
In post-anesthetic care unit			
Numeric rating scale, resting	4.1 ± 1.5	4.1 ± 1.6	>0.999
Numeric rating scale, active	6.2 ± 1.4	6.3 ± 1.3	0.749
The number of patients requesting for rescue analgesics	7 (30%)	7 (30%)	>0.999
Morphine equivalent dose of analgesics, mg	1.7 ± 3.1	1.6 ± 3.0	0.923
The number of patients receiving additional antiemetic agents	1 (4%)	1 (4%)	>0.999
Duration in the PACU, min	45 ± 16	47 ± 18	0.703
Postoperative 1–6 h			
Numeric rating scale, resting	4.1 ± 1.3	4.7 ± 1.3	0.116
Numeric rating scale, active	6.5 ± 1.3	6.7 ± 1.3	0.573
The number of patients requesting for rescue analgesics	9 (39%)	16 (70%)	0.075
Morphine equivalent dose of analgesics, mg	1.8 ± 2.3	3.1 ± 2.1	0.059
The number of patients receiving additional antiemetic agents	1 (4%)	1 (4%)	>0.999
Postoperative 6–24 h			
Numeric rating scale, resting	3.8 ± 1.2	4.0 ± 1.9	0.584
Numeric rating scale, active	5.9 ± 1.2	6.1 ± 1.5	0.593
The number of patients requesting for rescue analgesics	9 (39%)	13 (57%)	0.376
Morphine equivalent dose of analgesics, mg	1.8 ± 2.3	2.8 ± 2.7	0.189
The number of patients receiving additional antiemetic agents	3 (13%)	1 (4%)	0.608
Postoperative 24–48 h			
Numeric rating scale, resting	3.1 ± 1.8	3.3 ± 2.0	0.757
Numeric rating scale, active	5.2 ± 1.7	5.1 ± 1.8	0.869
The number of patients requesting for rescue analgesics	8 (35%)	10 (43%)	0.763
Morphine equivalent dose of analgesics, mg	1.8 ± 2.7	2.4 ± 3.0	0.528
The number of patients receiving additional antiemetic agents	0 (0%)	2 (9%)	0.489
Time to first passing of gas, h	80.7 ± 21.0	72.8 ± 14.9	0.145
Length of postoperative hospital stay, d	5 (5–11)	5 (5–8)	0.150
Satisfaction of surgeon			0.014 *
2	3 (13%)	0 (0%)	
3	7 (30%)	2 (9%)	
4	11 (48%)	12 (52%)	
5	2 (9%)	9 (39%)	
Postoperative complications			0.200
Pleural effusion	3 (13%)	0 (0%)	
Intraperitoneal infection	1 (4%)	1 (4%)	
Ileus	0 (0%)	1 (4%)	

Values are presented as mean ± SD or number of patients (percentage). NMB: neuromuscular blockade; PACU: post-anesthetic care unit. * *p* < 0.05.

## Data Availability

The datasets used and/or analyzed during the current study are available from the corresponding author upon reasonable request.
